# Plasma volume fraction in drug-resistant epilepsy: A DCE-MRI marker of microvascular pathology

**DOI:** 10.1016/j.nicl.2026.104044

**Published:** 2026-07-27

**Authors:** Amirhossein Talebanpour, Sheida Mirloo, Alireza Aleali, Lyna Kamintsky, Yonatan Serlin, Nir Cafri, Ilan Goldberg, Chris Bowen, Gal Ben-Arie, Theodor Rüber, Felix Benninger, Ben Whatley, Alon Friedman

**Affiliations:** a Department of Medical Neuroscience, Dalhousie University, Halifax, Nova Scotia, Canada; b The Departments of Physiology & Cell Biology, Cognitive & Brain Sciences, Professor Vladimir Zelman Inter-Disciplinary Center of Brain Sciences, Ben-Gurion University of the Negev, Beer-Sheva, Israel; c Department of Neurology, Rabin Medical Center, Beilinson Hospital and Tel-Aviv University, Petah Tikva, Israel; d Department of Diagnostic Radiology, Dalhousie University, Halifax, Nova Scotia, Canada; e Department of Medical Imaging, Soroka Medical Center, Beer Sheva, Israel; f Department of Neuroradiology, University Hospital Bonn, Bonn, Germany; g Department of Epileptology, University Hospital Bonn, Bonn, Germany; h German Center for Neurodegenerative Diseases, Bonn, Germany; i Center for Data Usability and Translation, University of Bonn, Bonn, Germany; j Division of Neurology, Dalhousie University, Halifax, Nova Scotia, Canada

**Keywords:** Plasma volume fraction (vₚ), Drug-resistant epilepsy (DRE), Blood-brain barrier dysfunction (BBBD), Dynamic contrast-enhanced magnetic resonance imaging (DCE-MRI), Epileptogenic zone localization, Microvascular remodeling

## Abstract

**Objectives:**

Microvascular remodeling and blood–brain barrier dysfunction (BBBD) are increasingly recognized as contributors to epilepsy. However, commonly used vascular imaging markers are often state-dependent and lack spatial specificity. We aimed to (1) validate plasma volume fraction (vₚ) derived from dynamic contrast-enhanced magnetic resonance imaging (DCE-MRI) as a sensitive marker of microvascular changes; (2) characterize the vₚ alterations in patients with drug-resistant epilepsy (DRE); and (3) assess the spatial relationship between vₚ abnormalities and BBBD.

**Methods:**

We analyzed DCE-MRI images from 49 people with epilepsy (PWE) and 68 healthy controls across two sites. vₚ and BBB permeability were quantified using BBBdetect software*.* Voxel-wise vₚ was estimated using the extended Tofts model, and BBBD was quantified using slope-based permeability mapping. Both measures were summarized using modified z-scores, with suprathreshold abnormality defined as modified z-score > 2. We performed region-wise and lobe-wise analyses restricted to gray matter and trained supervised classifiers to distinguish PWE from controls using regional z-vₚ and z-BBBD features.

**Results:**

Compared with controls, PWE showed increased voxel-wise and region-wise vₚ abnormality burden, with a non-uniform spatial pattern that includes prominent fronto-temporal elevations and frequent involvement of limbic regions. BBBD was common and spatially diffuse. Restricting analysis to regions with co-occurring suprathreshold vₚ and BBBD reduced spatial diffuseness relative to BBBD alone. Multivariate classification achieved encouraging test performance using vascular and barrier features (balanced accuracy = 0.81), with feature importance suggesting complementary contributions from vₚ and BBBD.

**Conclusion:**

vₚ is a sensitive DCE-MRI-derived marker of microvascular abnormalities in DRE. Integrating vₚ with BBBD enhances the spatial specificity of abnormality patterns and shows encouraging concordance with the clinically suspected epileptogenic territories, warranting prospective validation against clinical reference standards.

## Highlights

• Extended Tofts-derived vₚ captures microvascular abnormalities in DRE.
• Elevated vₚ in epilepsy may reflect angiogenesis-linked remodeling.
• vₚ restricts diffuse BBBD patterns into more focal vascular signatures.
• Machine learning supports the importance of vascular-barrier features.
• Joint vₚ-BBBD mapping may guide hypothesis-generating epileptogenic zone localization.

## 1 Introduction

Cerebral vascular integrity is fundamental to brain function, and subtle alterations in the microvasculature can provide valuable insight into neurological dysfunction and the mechanisms that drive disease (Van Lanen et al., 2021; Gorelick et al., 2011; Wardlaw et al., 2019). However, microvascular alterations are often subtle and are not easily captured by routine clinical imaging. Many commonly used imaging indicators of vascular status in clinical neuroscience are dominated by hemodynamic measures such as cerebral blood flow (CBF) and cerebral blood volume (CBV) (Fantini et al., 2016; Vu et al., 2024). However, CBF and CBV are highly sensitive to acute physiological and systemic fluctuations (e.g., vascular tone, blood pressure, PaCO₂), which can confound more chronic, subtle changes of the microvascular network (Clement et al., 2018; van der Kleij et al., 2020; Takahara et al., 2018).

Epilepsy has increasingly been linked to microvascular remodeling, angiogenesis, and blood-brain barrier dysfunction (BBBD) (Van Lanen et al., 2021; Rigau et al., 2007; Heinemann et al., 2012). Experimental studies suggest that microvascular alterations participate in epileptogenic mechanisms rather than occurring solely as downstream consequences (Van Lanen et al., 2021). In epileptogenic regions, recurring seizures and associated metabolic/inflammatory stressors can promote angiogenesis (Rigau et al., 2007). This remodeling may coincide with structurally immature or otherwise altered barrier properties, resulting in increased permeability (Van Lanen et al., 2021). Consistent with this framework, BBBD has been reported in epilepsy and is increasingly viewed as a measurable feature of epileptogenic pathology (Van Lanen et al., 2021; Rigau et al., 2007). Dynamic contrast-enhanced MRI (DCE-MRI) enables in vivo characterization of BBB leakage and related vascular parameters, providing a noninvasive window into these abnormalities (Chassidim et al., 2013; Veksler et al., 2014).

This perspective is particularly relevant in drug-resistant epilepsy (DRE), where surgery can be highly effective, but depends on accurate presurgical localization of the epileptogenic zone (Banerjee et al., 2014; Baumgartner et al., 2019). We have recently shown that BBB permeability maps derived from DCE-MRI can reveal abnormalities (Cafri et al., 2025); however, permeability patterns may be spatially diffuse and not always sufficiently specific to delineate the epileptogenic zone. Here we introduce plasma volume fraction (vₚ), a DCE-MRI–derived parameter reflecting the intravascular plasma/vascular volume contribution within a voxel, hypothesized to be sensitive to microvascular remodeling (e.g., altered microvascular density and/or angiogenesis). We quantify vₚ abnormalities in DRE and examine their spatial relationships to BBBD metrics in comparison with healthy controls.

## 2 Materials and methods

### 2.1 Human participants and ethics

Ethical approval for this study was obtained from the Ethics Committee at Soroka Medical Center in Beer Sheva, Israel (SUMC), as well as from the Nova Scotia Health Authority and Dalhousie University in Halifax, Canada (DAL). All participants provided written informed consent. The study was conducted in accordance with the Declaration of Helsinki and included only individuals aged 18 years or older.

### 2.2 Healthy controls

A total of 68 healthy individuals were recruited to establish the normative baseline for vascular parameters against which patients were compared (mean age ± standard deviation (SD) = 33.31 ± 10.78; range 19–75; 39.71% female). Of these, 25 were scanned at the DAL site and 43 at SUMC. Inclusion criteria were: (1) age 18–90 years; and (2) no known history of neurological, neurosurgical, or psychiatric disorders, with normal renal function. Individuals with contraindications to MRI were excluded.

### 2.3 Internal validation cases (stroke and tumor)

As an internal validation step, we examined vascular parameter maps in representative non-epilepsy pathology cases, including two brain tumor patients and one acute ischemic stroke patient. These cases were used to qualitatively and quantitatively verify that the derived maps captured lesion-associated vascular alterations and plausible lesion-to-perilesional gradients. The stroke case was drawn from a dataset previously reported in earlier work (Serlin et al., 2019).

### 2.4 Patients with epilepsy

Among the 49 participants (mean age ± SD = 32.71 ± 13.27; range 19–67; 46.94% female), 21 were scanned at the DAL site and 28 at SUMC. Patients were recruited consecutively during routine follow-up visits at the epilepsy clinics and represented a range of clinically suspected phenotypes, including temporal, frontal, and generalized-onset epilepsy (Table 1; detailed patient-level clinical-radiological characteristics are provided in Supplementary Table 1). These lesion categories were based on available clinical MRI and presurgical evaluation records. Inclusion criteria were: (1) age 18–90 years; (2) a clinical diagnosis of epilepsy based on the International League Against Epilepsy (ILAE) 2017 classification (Fisher et al., 2017); (3) at least one electroencephalography (EEG) study interpreted by an epileptologist; and (4) drug-resistant epilepsy (DRE), defined as ongoing seizures despite adequate trials of ≥2 appropriately chosen and tolerated antiseizure medications. Patients with renal impairment, contraindications to MRI contrast, or prior resective epilepsy surgery were excluded.

**Table 1:** Demographics.

	Controls *N* = 68	PWE *N* = 49	Temporal *N* = 19	Frontal *N* = 13	Generalized *N* = 10	Others *N* = 7
Age, years (SD)	33.31 (10.79)	32.71 (13.27)	31.59 (11.84)	39.38 (15.71)	30.46 (13.95)	38.42 (12.85)
Sex, female N (%)	27 (39.71)	23 (46.94)	7 (36.84)	7 (53.85)	6 (60.00)	3 (42.86)
Lesional, N (%)		16 (32.65)	7 (36.84)	6 (46.15)	3 (30.00)	0 (0.00)

Others comprised fronto-temporal (n = 1), limbic (n = 2), parietal (n = 2), temporo-occipital (*n* = 1), and left-sided non-lobar (n = 1).

### 2.5 Magnetic resonance imaging

All participants underwent scanning on 3 T MRI systems (SUMC: Philips Ingenia; DAL: General Electric). Scanning was done at least 72 h after the last reported seizure. The image acquisition protocol followed the previously described methods (Cafri et al., 2025; Serlin et al., 2019; Veksler et al., 2020). Briefly, T1- and T2-weighted imaging, FLAIR, and DESPOT1 were acquired, followed by a DCE sequence. After acquisition of the pre-contrast dynamic baseline, a gadolinium-based contrast agent was administered intravenously (SUMC: Dotarem; relaxivity 3.89 L·mmol^−1^·s^−1^; DAL: MultiHance; relaxivity 6.3 L·mmol^−1^·s^−1^).

### 2.6 v_p_ analysis

Vascular analysis was performed using the BBBdetect software *(Emagix Inc, NS, Canada)* (Veksler et al., 2020; Kamintsky et al., 2020). All images were registered and normalized to Montreal Neurological Institute (MNI) space using Statistical Parametric Mapping (SPM)12 before analysis (SPM, 2026). The extended Tofts model was used to estimate vₚ (Tofts, 1997; Sourbron and Buckley, 2011). Briefly, dynamic signal intensity-time courses were converted to contrast-agent concentration-time curves using baseline T1 information and the appropriate relaxivity for each contrast agent (Deoni et al., 2005). An arterial input function (AIF) was extracted from a region of interest in the middle cerebral artery (MCA) to obtain the arterial plasma concentration–time curve (C_p_(t)). Voxel-wise tissue concentration curves, C_t_(t), were fit to the extended Tofts formulation, which explicitly includes an intravascular plasma term:

(1) $$C_{t}\left(t\right)=v_{p}C_{p}\left(t\right)+K^{\text{trans}}\int_{0}^{t}C_{p}\left(\tau \right)e^{-k_{\mathrm{ep}}\left(t-\tau \right)}\;\mathrm{d\tau}$$

where Cₜ(t) and Cₚ(t) are the tissue and arterial plasma contrast-agent concentration–time curves, respectively; vₚ is the fractional plasma volume; K^trans^ reflects contrast-agent transfer from plasma to the extravascular extracellular space; kₑₚ is the return rate constant from the extravascular extracellular space to plasma (Tofts, 1997).

After voxel-wise vₚ mapping, raw vₚ values were retained for quantitative summaries. Because vₚ estimation during DCE-MRI is influenced by T1-dependent signal conversion, differences in scanner characteristics and acquisition parameters can shift baseline quantitative values (see Fig. S1 for better illustration). We therefore used site-specific control groups to define the expected regional vₚ distribution, and values were transformed into modified z-scores relative to their corresponding control cohort.

For inter-site case-control abnormality mapping, voxel-wise vₚ was converted to a robust, control-referenced modified z-score using the control median and median absolute deviation (MAD). Suprathreshold abnormalities were defined as z-v_p_ > 2, a robust statistical cutoff for identifying substantial positive deviations from a control-referenced distribution (Leys et al., 2013; Verdi et al., 2023). Region-level analyses were performed by segmenting the MNI-normalized brain into 120 atlas regions (Neurodebian, 2026); voxels/regions meeting the suprathreshold criterion were flagged and summarized as regional abnormality burden for group comparisons. Because abnormal regions showed limited overlap across individual patients, and region-wise testing across 120 atlas regions would require extensive multiple-comparison correction, inferential analyses were performed at the level of 9 atlas-defined lobes. This approach was used to capture broader, more convergent patterns of abnormality.

### 2.7 BBB analysis

Semi-quantitative BBBD was assessed using BBBdetect software *(Emagix Inc, NS, Canada)* as reported previously (Chassidim et al., 2013; Veksler et al., 2014; Cafri et al., 2025; Veksler et al., 2020). Permeability maps were generated by fitting a linear model to concentration-time curves and calculating the slope from 6 min post-injection to the final DCE volume. Regional slopes were normalized to the superior sagittal sinus to account for interindividual variability in intracranial contrast flow. For control comparisons, regional slopes were converted to control-referenced modified z-scores using the control median and MAD, and regions with z-BBBD >2 were marked as showing BBBD.

### 2.8 Radiomics feature selection and machine learning

Supervised machine-learning classification models were employed to evaluate how well regional vascular features differentiate PWE from healthy controls. Regional features were extracted by computing control-referenced modified z-scores for vₚ (z-vₚ) and BBBD (z-BBBD) in each of the 120 atlas regions. Infrequent region-level missing values (5.5% of the entire feature matrix) arose from insufficient valid voxels or unsuccessful vₚ/BBBD estimation within individual parcels and were handled by median imputation (Berkelmans et al., 2022). The feature matrix was refined using L1-regularized regression (LASSO), with the regularization parameter optimized via cross-validation (α = 0.001–0.1), retaining features with non-zero coefficients (Tibshirani, 1996). Highly correlated features were pruned (Pearson |r| > 0.85) (Kuhn, 2008). Data were partitioned into training and held-out test sets (70/30 stratified by group). Within the training set, model performance was evaluated using stratified 5-fold cross-validation, with feature standardization applied within each fold to prevent data leakage. Three classifiers were implemented, including logistic regression (LR), random forest (RF), and support vector machine (SVM). Hyperparameters were optimized using grid search within cross-validation, with balanced accuracy (BAC) as the primary objective to account for class imbalance (Brodersen et al., 2010). Model performance was quantified using accuracy, BAC, weighted F1-score, and area under the receiver operating characteristic curve (ROC-AUC), with final evaluation conducted on the independent test set. Feature importance was assessed using model-based measures to quantify the relative contribution of each predictor to classification. Model performance was further characterized by using confusion matrices for both training and test sets. Uncertainty in held-out test-set performance was estimated using 10,000 stratified bootstrap iterations of the fixed test-set predictions, with percentile-based 95% confidence intervals reported for each metric.

### 2.9 Statistical analysis

Continuous variables were summarized as median ± MAD, as appropriate to the non-normal distribution of the data. Group differences for continuous variables were assessed using nonparametric Mann–Whitney *U* tests. For paired within-subject comparisons, two-sided Wilcoxon signed-rank tests were used. For lobar analyses, multiple-comparison correction was performed using the Benjamini–Hochberg false discovery rate (FDR) procedure. For the tumor validation examples, lesion region-of-interest (ROI) versus peri-lesional shells were compared using Welch's two-sample *t*-tests, with Holm–Bonferroni correction applied across shell comparisons. Categorical variables were compared using the chi-square test or Fisher's exact test when expected cell counts were small. All tests were two-sided, and statistical significance was set at *p* < 0.05.

### 2.10 Spatial concordance and localization analysis

Exploratory qualitative localization analysis was performed in focal epilepsy cases (*n* = 39). For each patient, regional abnormality maps generated from each method (vₚ alone, BBBD alone, and the vₚ-BBBD overlap) were compared with the clinically suspected epileptogenic territory by a neuroscientist. Clinical suspicion was determined from the available clinical investigation data, including seizure semiology, EEG findings, and structural MRI when a lesion was present. Cases were assigned to one of five predefined qualitative localization categories based on the spatial relationship between the imaging abnormality and the suspected epileptogenic territory (Table 2). For secondary summary analysis, categories A and B were further grouped as localizing, whereas categories C, D, and E were grouped as non-localizing (Bitzer et al., 2024).

**Table 2:** Qualitative localization categories.

Category	Label	Interpretation
A	Focal concordant	Joint vascular abnormality focally matches the clinically suspected epileptogenic lobe or region
B	Concordant, non-focal	Joint vascular abnormality overlaps the suspected epileptogenic territory, is spatially broader than a discrete focal match
C	Ipsilateral discordant	Joint vascular abnormality is located on the same side as the suspected focus, but outside the suspected lobe(s)
D	Widespread	Joint vascular abnormality is broad, multilobar, bilateral, or diffuse, preventing focal localization
E	No suprathreshold abnormality	No significant joint vascular abnormality is identified

Spatial-null sensitivity analysis was performed using the BrainSpace implementation of Moran spectral randomization (Vos de Wael et al., 2020). For each focal epilepsy case, the clinically suspected epileptogenic territory was mapped to atlas-defined regions based on lobe and laterality. The observed joint vₚ-BBBD abnormality map was compared with 1000 spatially constrained surrogate maps preserving regional spatial autocorrelation. The *p*-value was calculated as the proportion of surrogate maps with a localizing rate equal to or greater than the observed rate.

## 3 Results

### 3.1 Normative v_p_ reference and method validation

**Fig. 1:**
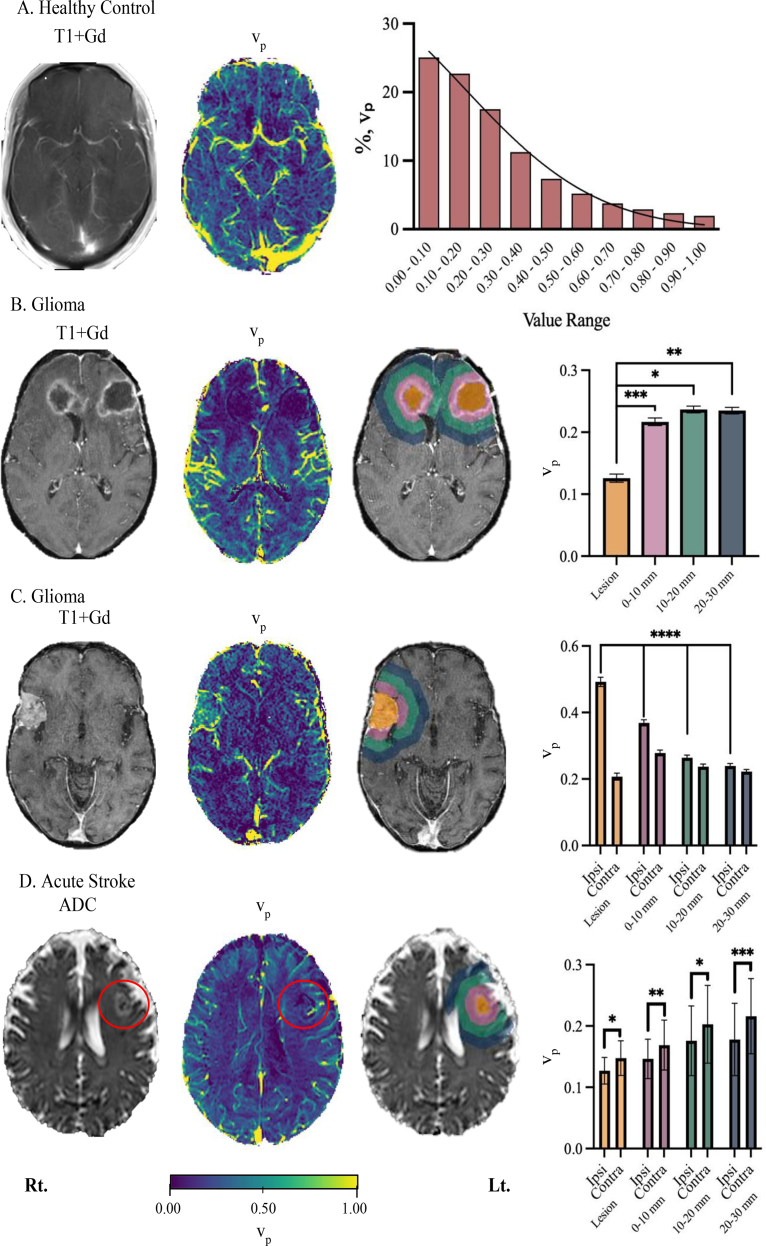
**Representative anatomical MRI and plasma volume fraction (vₚ) maps across conditions.** A. Healthy control: T1-weighted post-contrast (T1 + Gd) image and corresponding vₚ map, with the voxel-wise histogram illustrating the right-skewed distribution. B. Malignant glioma case 1: T1 + Gd image, vₚ map, and lesion-centered non-overlapping distance shells (0–10, 10–20, 20–30 mm); bar plot shows voxel-wise mean ± SD vₚ in lesion ROI and shells; C. Malignant glioma case 2: T1 + Gd image, vₚ map, and distance shells; bar plot shows mean ± SD vₚ for ipsilateral vs mirrored contralateral tissue (For B—C lesion vs each shell in ipsilateral side was tested using two-sided Welch's two-sample *t*-test with Holm-Bonferroni correction; ipsilateral-contralateral differences were quantified for the lesion ROI and each shell; Lesion: *p* < 0.0001; 0–10 mm: *p* < 0.0001; 10–20 mm: *p* < 0.02; 20–30 mm: *p* = ns; these *p-*values are not annotated on the plot) D. Acute ischemic stroke case: ADC image, vₚ map, and distance shells; bar plot shows median ± MAD vₚ for ipsilateral vs mirrored contralateral tissue (ipsilateral vs contralateral comparisons were tested using paired Wilcoxon signed-rank tests on matched voxel subsamples). * *p* < 0.05, ** *p* < 0.01, *** *p* < 0.001, **** *p* < 0.0001. MRI, magnetic resonance imaging; vₚ, plasma volume fraction; Gd, gadolinium; SD, standard deviation; ROI, region-of-interest; ADC, Apparent Diffusion Coefficient; MAD, median absolute deviation; Rt, right; Lt, left.

vₚ maps derived from healthy scans showed spatial patterns consistent with vascular anatomy on T1-weighted post-contrast images, with the highest vₚ values localized to visible blood vessels and other vascular-rich regions, rather than widespread parenchymal distribution (Fig. 1A), supporting vₚ as an imaging-derived marker of cerebral plasma volume. Across 25 young healthy controls (mean age ± SD: 41.7 ± 13.5 years; range 20–75; 6 females), voxel-wise vₚ values exhibited a right-skewed distribution, with most values concentrated at the low end and a diminishing upper tail toward higher vₚ (Fig. 1A).

To further validate our v_p_ measurements, we next analyzed scans from patients with acute ischemic stroke and brain tumors, two conditions with well-characterized microvascular changes (Fig. 1B-D). Across two representative tumor cases, shell-based vₚ analysis revealed marked lesion-associated heterogeneity, with significant differences between the lesion ROI and peri-lesional shells (0–10, 10–20, and 20–30 mm; Fig. 1B-C). The first case showing central necrosis and ring enhancement revealed corresponding reduced vₚ within the core relative to all shells (*p* < 0.05). In the second case, the tumor core showed contrast enhancement and correspondingly higher vₚ with clear hemispheric asymmetry compared with mirrored contralateral tissue (*p* < 0.0001). In a representative acute stroke case, vₚ was significantly lower on the ipsilateral (lesion – see ADC map in Fig. 1D) side compared with the mirrored contralateral hemisphere (*p* < 0.05; Fig. 1D).

### 3.2 Voxel-wise vₚ abnormality burden is higher in PWE

**Fig. 2:**
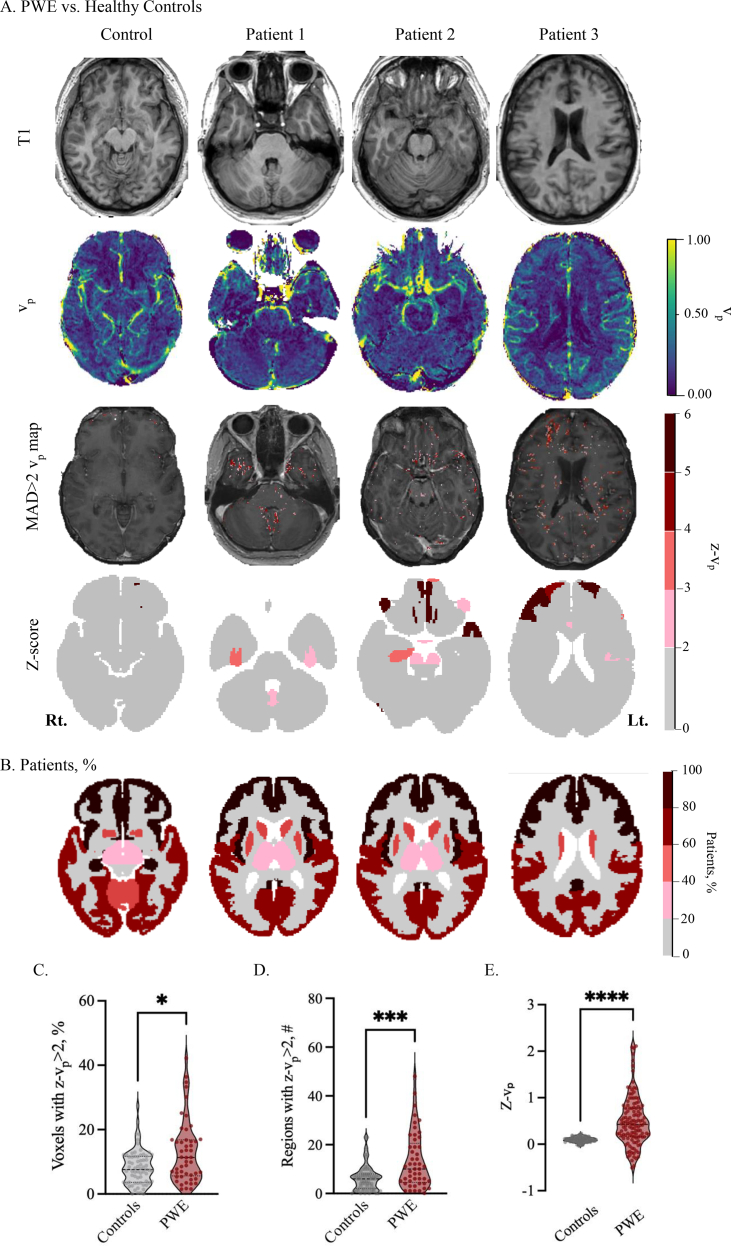
**Elevated plasma volume fraction (vₚ) abnormalities in people with epilepsy (PWE) relative to healthy controls.** A. Representative axial slices illustrating anatomical T1-weighted images (top row), corresponding voxel-wise vₚ maps (second row; color scale 0–1), and control-referenced vₚ modified z-score maps. The voxel-wise vₚ heatmaps use a 0–1 color scale, where yellow indicates higher raw vₚ values. Abnormality maps are thresholded at z-vₚ > 2; pink-to-red colors denote increasing suprathreshold z-vₚ modified z-score values, while gray indicates non-suprathreshold brain tissue and white indicates ventricles/background. From left to right: a healthy control, male, 23 years; a PWE with right mesial temporal epilepsy, female, 24 years; a PWE with frontal/midline epilepsy, male, 29 years; a PWE with suspected frontal epilepsy, female, 65 years. Suprathreshold voxels were defined as z-vₚ > 2 and visualized as abnormality overlays superimposed on anatomy (third row), with the corresponding thresholded z-vₚ maps shown below (fourth row; color scale indicates z-vₚ modified z-score values). B. Lobe-level prevalence map summarizing the proportion of PWE with suprathreshold abnormality (z-vₚ > 2) within each lobe/compartment (color scale denotes % of patients). C. Subject-level voxel abnormality burden, quantified as the percentage of brain voxels with z-vₚ > 2, is increased in PWE (*n* = 49) relative to controls (*n* = 68); two-sided Mann-Whitney *U* test (*p* = 0.0118). D. Subject-level regional abnormality burden, quantified as the number of regions with z-vₚ > 2, is higher in PWE (n = 49) than controls (n = 68); two-sided Mann-Whitney U test (*p* = 0.0006). E. Regional analysis across the 120 atlas regions showing higher z-vₚ values in PWE compared with controls (each point represents the median z-vₚ for one region per group); group difference tested using a Wilcoxon test (*p* < 0.0001). Dashed horizontal lines indicate the median and interquartile range. Asterisks denote significance (* *p* < 0.05, ** *p* < 0.01, *** *p* < 0.001, **** *p* < 0.0001). PWE, people with epilepsy; vₚ, plasma volume fraction; Rt, right; Lt, left.

Building on the normative reference and validation findings, standardized vₚ mapping was next applied to epilepsy to quantify patient-specific abnormalities. There were no significant differences found in age (*p* = 0.86) or sex (*p* = 0.31) between PWE and the controls. Demographics for all PWE are presented in Table 1. vₚ maps generated for each patient were superimposed on the T1-weighted anatomical images, highlighting voxels with a vₚ modified z-score (z-vₚ) > 2 relative to controls (Fig. 2A). PWE had a higher median percentage of brain voxels with z-vₚ > 2 compared with controls (median ± MAD: 11.33% ± 5.59 vs 7.59% ± 4.02, *p* = 0.0118; Fig. 2C).

### 3.3 Regional analysis

We next tested the regional distribution of elevated vₚ in PWE. Because absolute vₚ values were not directly comparable across sites, subsequent abnormality analyses were based on site-specific control-referenced modified z-scores. Directional assessment of suprathreshold vₚ abnormalities showed that elevated vₚ was the predominant pattern in PWE. Across patient-region observations, 475 patient-region observations showed z-vₚ > 2, whereas only 72 showed z-vₚ < −2. Consistently, all patients showed elevated vₚ in at least one region, while only 10 patients showed reduced vₚ in at least one region. Using the modified z-score approach, PWE showed a greater burden of regional vₚ abnormality, with more regions exceeding the suprathreshold cutoff of z-vₚ > 2 than controls (median ± MAD: 10 ± 8 vs 6 ± 3; *p* = 0.0006; Fig. 2D). Site-matched comparisons of PWE versus their corresponding control cohorts were also performed separately for SUMC and DAL as a sensitivity analysis and are provided in Supplementary Materials (Fig. S4). Elevated vₚ was most frequently observed in the right orbital part of the inferior frontal gyrus (36.74% of patients), left middle temporal gyrus (34.69%), left central operculum (32.65%), right opercular part of the inferior frontal gyrus (32.65%), and right postcentral gyrus (32.65%) (Table S2, Fig. S2). However, despite the overall increase in regional z-vₚ values in PWE, only three regions reached the cutoff of z-vₚ > 2 at the group level (Fig. 2E). No region reached the reduced-abnormality threshold at the group level (z-vₚ < −2). We therefore next examined lobe-level patterns of vₚ abnormality.

The percentage of patients showing elevated vₚ in at least one region within each lobe was highest in the frontal lobe (97.98% vs 27.94% in controls), followed by the limbic system (87.76% vs. 32.35%), temporal (77.55% vs. 26.47%), and occipital (73.46% vs. 16.65%) (Table 3, Fig. 2B).

**Table 3:** Lobe-wise vₚ abnormalities.

Lobe	%, Patients	%, Controls	Site	v_p_, Patients	v_p_, Controls	*p*-value (uncorrected)
**Frontal**	97.98	27.94	*SUMC*	0.1369	0.1194	0.0194
			*DAL*	0.2618	0.2242	0.0412
**Limbic**	87.76	32.35	*SUMC*	0.1055	0.1007	0.2681
			*DAL*	0.2600	0.2351	0.0814
**Temporal**	77.55	26.47	*SUMC*	0.1327	0.1111	0.0275
			*DAL*	0.2784	0.2338	0.0249
**Occipital**	73.46	16.65	*SUMC*	0.1207	0.1208	0.9116
			*DAL*	0.2933	0.2911	0.8067
**Parietal**	65.35	19.12	*SUMC*	0.1107	0.1095	0.5786
			*DAL*	0.3190	0.2949	0.9131
**Cerebellum**	48.98	17.65	*SUMC*	0.0873	0.0943	0.4093
			*DAL*	0.2692	0.2407	0.7267
**Basal Ganglia**	42.85	19.12	*SUMC*	0.0913	0.0890	0.2249
			*DAL*	0.2375	0.2119	0.0703
**Diencephalon**	32.65	8.82	*SUMC*	0.0832	0.0733	0.7568
			*DAL*	0.2290	0.2017	0.4439
**Brain Stem**	14.29	7.35	*SUMC*	0.0680	0.0695	0.6028
			*DAL*	0.1972	0.1903	0.6307

Site-specific median lobar vₚ comparisons were examined as a secondary analysis. Before multiple-comparison correction, several lobes showed nominal group differences between PWE and controls, including higher frontal and temporal vₚ in PWE at both sites, and higher limbic vₚ in PWE at DAL. However, after correction, none of the site-specific lobar comparisons remained statistically significant (Table 3).

### 3.4 Epilepsy phenotype analysis

When v_p_ values were compared according to the type of epilepsy, we found that the generalized epilepsy group had a higher regional vₚ abnormality burden than the focal epilepsy cohort, with a higher number of regions exceeding z-vₚ > 2 (median ± MAD: 18 ± 9.5 vs 7 ± 7.5; *n* = 10 vs 39; *p* = 0.0095; Fig. S3A). However, no corresponding difference was observed at the voxel level, where the percentage of voxels with z-vₚ > 2 was comparable between generalized and focal epilepsy (median ± MAD: 14.19% ± 12.5 vs 10.04% ± 5.14, *p* = 0.5581; Fig. S3B).

Because only one patient in the DAL cohort was classified as generalized epilepsy, lobar phenotype analysis was performed in the SUMC cohort only. In the SUMC cohort, raw median lobar vₚ was compared between generalized and focal epilepsy. Before multiple-comparison correction, significantly higher vₚ was observed in generalized epilepsy within the frontal lobe and parietal lobe. However, none of these differences remained statistically significant after correction for multiple comparisons (Table 4). In focal epilepsy patients, no significant differences in vₚ were found between suspected frontal and temporal lobe epilepsy groups.

**Table 4:** Lobe-wise vₚ by epilepsy phenotype in the SUMC cohort.

Lobe	v_p_, Generalized	v_p_, Focal	*p*-value (uncorrected)
Frontal	0.1571	0.1112	0.0325
Parietal	0.1536	0.1023	0.0345
Cerebellum	0.1164	0.0817	0.0539
Brain Stem	0.0859	0.0655	0.4385
Occipital	0.1346	0.1021	0.5950
Basal Ganglia	0.0874	0.0879	0.6993
Limbic	0.1050	0.0981	0.7355
Diencephalon	0.0868	0.0820	0.8849
Temporal	0.1319	0.1140	0.9231

### 3.5 Lesional vs. non-lesional

Within the PWE cohort, 16 patients were classified as MRI-positive (visible structural lesion on clinical MRI), while 33 were MRI-negative. In most MRI-positive cases, vₚ abnormality appeared more prominent in the hemisphere ipsilateral to the lesion than in the contralateral hemisphere (11 vs. 3; two cases had bilateral lesions).

**Fig. 3:**
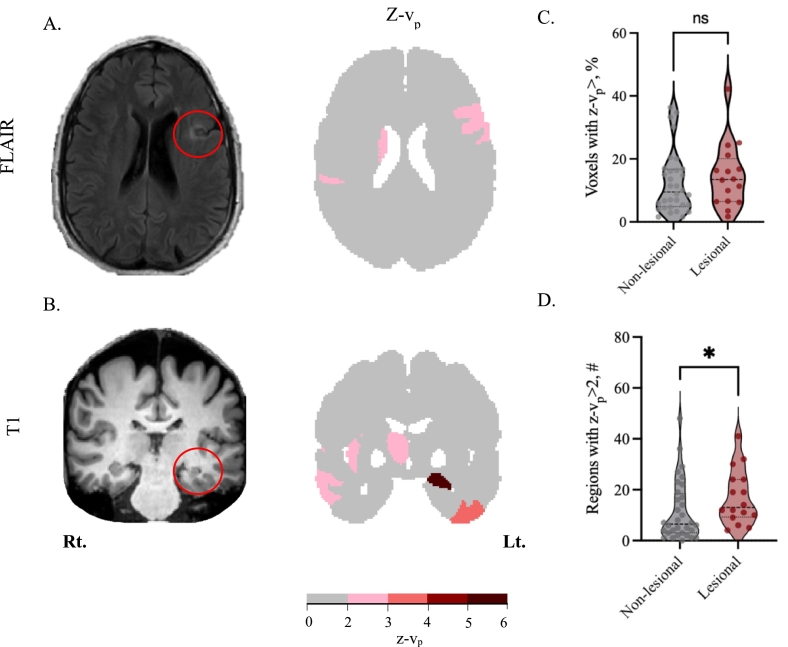
**Lesion-associated versus non-lesional vₚ abnormality burden in PWE.** (A–B) Representative examples of lesional PWE illustrating anatomical MRI images (left) and corresponding control-referenced vₚ abnormality maps (right) derived from control-referenced vₚ modified z-scores (z-vₚ). Panel A shows an axial FLAIR image; Panel B shows a coronal T1-weighted image. Abnormality maps are thresholded at z-score > 2 and displayed on a standardized brain template; pink-to-red colors denote increasing suprathreshold z-vₚ modified z-score values, while gray indicates non-suprathreshold brain tissue and white indicates ventricles/background. (A) Male, 33 years, frontal lobe epilepsy; structural lesion characterized by cortical irregularity involving the left precentral gyrus, probably representing focal cortical dysplasia (FCD) and extending to the left inferior frontal gyrus. (B) Male, 35 years, focal epilepsy with focal-to-bilateral tonic-clonic seizures (FBTCS); structural lesion consistent with left mesial temporal sclerosis. (C) Voxel-wise abnormality burden, quantified as the percentage of brain voxels exceeding threshold (z-vₚ > 2), did not differ between lesional (*n* = 16) and non-lesional PWE (*n* = 33); two-sided Mann–Whitney *U* test (*p* = 0.3797). (D) Region-wise abnormality burden, quantified as the number of atlas regions with z-vₚ > 2, was higher in lesional than non-lesional PWE; two-sided Mann–Whitney U test (*p* = 0.0322). Points represent individual subjects. Dashed horizontal lines indicate the median and interquartile range. “ns” indicates not significant; asterisk denotes significance (* *p* < 0.05). PWE, people with epilepsy; vₚ, plasma volume fraction; MRI, magnetic resonance imaging; FLAIR, Fluid-Attenuated Inversion Recovery; Rt, right; Lt, left.

At the group level, MRI-positive patients did not show a significantly greater voxel-wise burden of vₚ abnormality compared with MRI-negative patients (% voxels with z-vₚ > 2: median ± MAD: 13.44 ± 6.80 vs 9.45 ± 5.92; *p* = 0.3797; Fig. 3C). However, MRI-positive patients exhibited a higher region-wise abnormality burden, with more brain regions exceeding z-vₚ > 2 (median ± MAD: 13.00 ± 7.38 vs 6.50 ± 7.75; *p* = 0.0322; Fig. 3D).

Among MRI-positive cases, no significant difference in vₚ abnormality burden was observed between non-MTS lesions and MRI-suspected MTS at either the regional level (median ± MAD regions with z-vₚ > 2: 19.00 ± 8.00 vs. 9.00 ± 5.00; *p* = 0.1067) or voxel level (% voxels with z-vₚ > 2: 14.96 ± 6.23 vs. 7.20 ± 3.39; *p* = 0.1211).

### 3.6 | joint vascular signature reduces spatial diffuseness

We next asked to what extent v_p_ analysis corresponds to abnormal BBB permeability that has previously been reported as more common in PWE (Cafri et al., 2025). Building on the vₚ directionality analysis reported in 3.3, we assessed whether z-vₚ and z-BBBD abnormalities showed directional agreement across patient-region observations. Elevated abnormalities predominated for z-BBBD, with 1806 elevated (z-BBBD >2) patient-region observations and only 16 reduced (z-BBBD < −2). We then examined directional agreement within the two-tailed joint abnormality mask, defined as |z-vₚ| > 2 and |z-BBBD| > 2. A total of 228 patient-region observations showed joint suprathreshold abnormality, including 180 positive-positive, 0 negative-negative, 1 positive-negative, and 47 negative-positive observations. The directional agreement was 78.9% (180/228), driven primarily by positive-positive observations. We therefore defined a joint vascular signature using the intersection approach, focusing on regions simultaneously exceeding suprathreshold criteria for BBBD (z-BBBD >2) and plasma-volume abnormality (z-vₚ > 2).

**Fig. 4:**
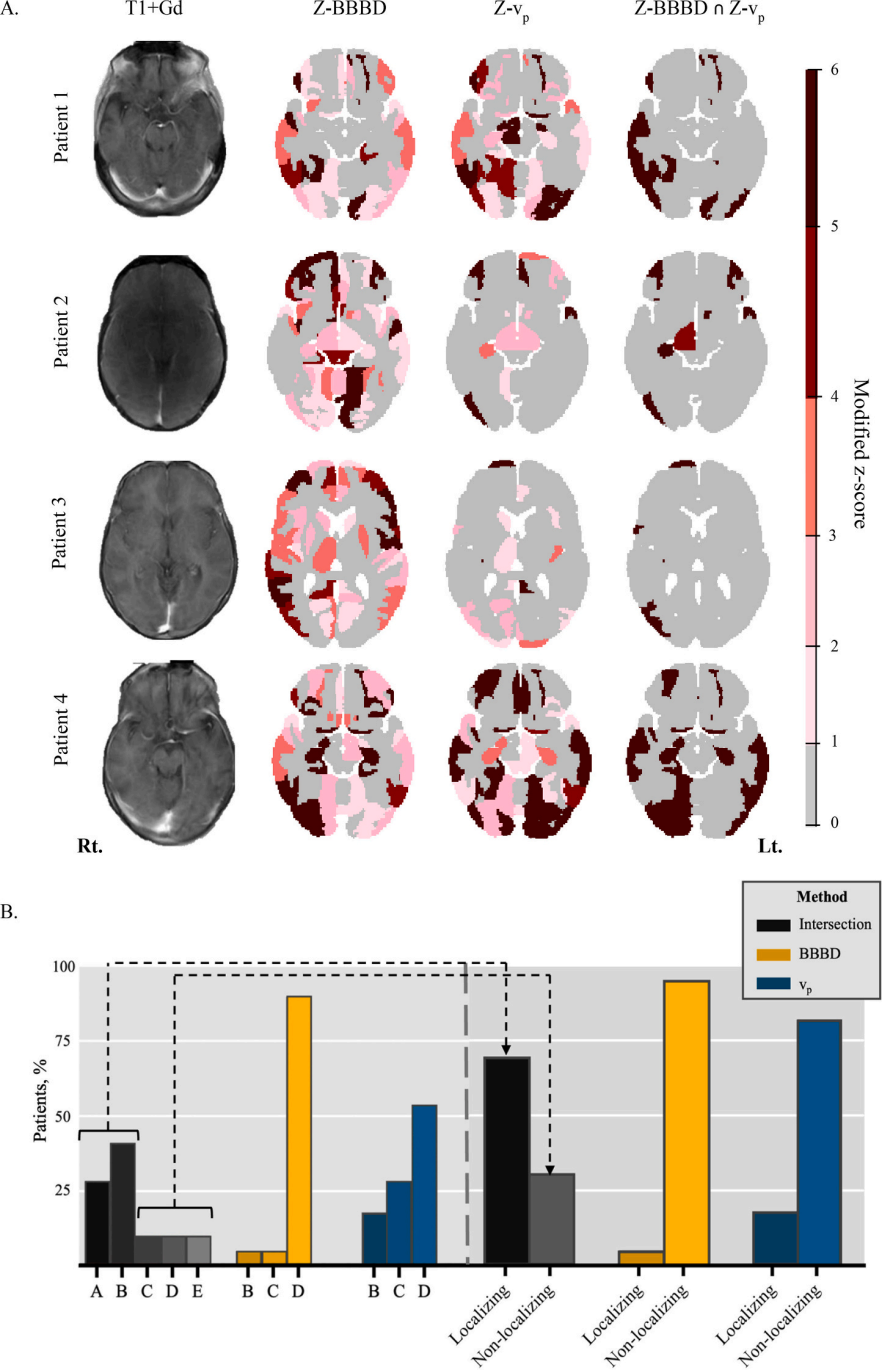
**Joint vascular abnormality metric highlights convergence of microvascular pathology (vₚ & BBBD) in epilepsy**. A. Representative axial slices showing (left-to-right) post-contrast T1-weighted images (T1 + Gd), control-referenced BBBD modified z-score maps (z-BBBD>2), (z-vₚ > 2) maps, and the intersection map identifying regions where both BBB and vₚ are high (z-BBBD >2 ∩ z-vₚ > 2). Abnormality maps are thresholded at z-vₚ > 2 and displayed on a standardized brain template; pink-to-red colors denote increasing suprathreshold modified z-score values, while gray indicates non-suprathreshold brain tissue and white indicates ventricles/background. From top to bottom, clinical diagnosis of PWE: right temporal epilepsy with impairment of consciousness, female, 20 years; bifrontal/midline epilepsy with focal-to-bilateral tonic–clonic seizures (FBTCS), male, 29 years; frontal lobe epilepsy with a left occipital lesion, female, 29 years; and generalized tonic–clonic epilepsy (GTCE), male, 65 years. B. Cohort-level qualitative localization analysis on focal epilepsy cases (*n* = 39) comparing the joint vascular signature (intersection), BBBD alone, and vₚ alone. Cases were classified into five categories: focal concordant (A), concordant non-focal (B), ipsilateral discordant (C), widespread (D), and no suprathreshold abnormality (E). The intersection method showed the highest proportion of localizing cases, with 28.2% classified as focal concordant and 41.0% as concordant non-focal, yielding an overall localizing rate of 69.2% when categories A and B were combined. BBBD alone was predominantly widespread (89.7%) and achieved localization in only 5.1% of cases, while vₚ alone was distributed across concordant non-focal (17.9%), ipsilateral discordant (28.2%), and widespread (53.8%) categories, with an overall localizing rate of 17.9%. For binary summary, categories A and B were considered localizing, whereas categories C, D, and E were considered non-localizing. Gd, gadolinium; BBBD, blood-brain barrier dysfunction; vₚ, plasma volume fraction; Rt, right; Lt, left.

Generally, z-BBBD maps were more diffuse compared to z-v_p_ maps (Fig. 4A). Restricting interpretation to the overlap between z-BBBD and z-vₚ reduced spatial diffuseness and highlighted a more spatially concentrated set of regions. At the regional level, 180 regions met both criteria. This overlap accounted for 9.97% of all BBBD-abnormal regions (180/1806) and 37.9% of all vₚ-abnormal regions (180/475).

As an exploratory approach to descriptively assess spatial concordance, we applied the qualitative localization framework to focal epilepsy cases. In this framework, the joint vascular signature showed the highest proportion of cases classified as concordant with the clinically suspected epileptogenic territory. When categories A and B (see Table 2) were grouped as localizing and categories C, D, and E as non-localizing, the intersection method achieved localization in 69.2% (27/39) of cases, showing a higher proportion of cases classified as localizing than BBBD alone or vₚ alone (Fig. 4B). In contrast, BBBD alone was classified as non-localizing in most patients, consistent with its broad and diffuse abnormality pattern, whereas vₚ alone showed an intermediate profile. Spatial-null sensitivity analysis further supported this concordance pattern. Using Moran spectral randomization, the observed localizing rate of the joint vₚ-BBBD signature was 71.8% (28/39), exceeding the mean localizing rate expected under the spatial null model (51.0%; 95% null interval: 41.0–61.5%; empirical *p* = 0.001; Fig. S7).

### 3.7 Machine learning performance

Supervised machine learning models (LR, RF, SVM) were trained to classify PWE versus controls using regional vascular features derived from an initial set of 240 variables. Following L1-based feature selection (α = 0.1), 33 features were retained for model training. Among the classifiers, LR showed the highest test-set BAC (0.81). Full performance metrics for all models are provided in Table 5. Feature-importance analysis of the LR model showed that both z-vₚ and z-BBBD features were prominently represented among the top-ranked predictors, with broadly comparable contributions across the retained feature set. Confusion matrices demonstrated similar classification patterns across training and test sets, with accurate identification of both controls and PWE groups (see supplementary figures for feature-importance analysis (Fig. S5) and confusion matrices (Fig. S6)).

**Table 5:** Machine learning performance.

**Model**	**Train**				**Test**			
	**Accuracy**	**BAC**	**F1-score**	**ROC-AUC**	**Accuracy, 95% CI**	**BAC, 95% CI**	**F1-score, 95% CI**	**ROC-AUC, 95% CI**
**LR**	**0.7294**	**0.7116**	**0.7230**	**0.7571**	**0.8333 [0.7222–0.9444]**	**0.8095 [0.6856–0.9333]**	**0.8278 [0.7046–0.9437]**	**0.8381 [0.6921–0.9619]**
RF	0.7912	0.7575	0.7702	0.7962	0.7778 [0.6667–0.8889]	0.7333 [0.6000–0.8667]	0.7552 [0.5926–0.8852]	0.7937 [0.6221–0.9238]
SVM	0.7162	0.7102	0.7130	0.7271	0.7500 [0.6389–0.8611]	0.7095 [0.5762–0.8429]	0.7298 [0.5718–0.8580]	0.8603 [0.7270–0.9619]

### 3.8 Medications

Since all patients were receiving antiseizure medication, vₚ abnormality burden was compared across medication groups. Because most patients were receiving polytherapy, medication groups were not mutually exclusive. No significant differences were observed for valproate (% voxels: median 4.959 vs 10.11; n = 17 vs 32; *p* = 0.0920; # regions: median 6.0 vs 11.5; *n* = 17 vs 32; *p* = 0.0781), carbamazepine (% voxels: median 14.96 vs 8.011; *n* = 9 vs 40; *p* = 0.1310; # regions: median 19.0 vs 9.5; n = 9 vs 40; *p* = 0.1056), levetiracetam (% voxels: median 8.475 vs 9.066; n = 17 vs 32; *p* = 0.5637; # regions: median 10.0 vs 10.5; n = 17 vs 32; *p* = 0.5917), or lacosamide (% voxels: median 5.785 vs 9.066; *n* = 7 vs 42; *p* = 0.7856; # regions: median 7.0 vs 10.5; *n* = 7 vs 42; *p* = 0.7422). However, lamotrigine users showed lower vₚ abnormality burden (% voxels: median 4.959 vs 11.48; *n* = 24 vs 25; *p* = 0.0063; # regions: median 6.0 vs 14.0; n = 24 vs 25; *p* = 0.0064).

## 4 Discussion

We found that vₚ maps behaved as expected in validation conditions with established microvascular pathology, showing lesion-to-perilesional gradients in tumors and reduced ipsilateral vₚ in acute ischemic stroke. In epilepsy, PWE showed a greater burden of microvascular pathology (elevated vₚ) than controls, with prominent fronto-temporal effects and limbic involvement. MRI-positive cases tended to show clustered abnormalities in the hemisphere ipsilateral to the lesion. Finally, combining regional vₚ and BBBD features improved discrimination of PWE versus healthy controls, supporting the value of integrating structural/volumetric (v_p_) and functional (BBBD) microvascular measures for distinguishing PWE from controls and hypothesis-generating localization.

A substantial body of human MRI work has demonstrated BBBD in epilepsy using quantitative contrast-enhanced scans (Cafri et al., 2025; Rüber et al., 2018; Reiter et al., 2024; Schulte et al., 2024). In parallel, studies have used perfusion-sensitive MRI measures to support seizure-focus localization and to characterize peri-ictal physiopathology (Gaxiola-Valdez et al., 2017; Kim et al., 2016; Nagesh et al., 2018). However, the integration of a direct intravascular volume measure with BBBD as complementary markers of structural and functional microvascular pathology in epilepsy has not yet been established in human MRI studies. Our finding of elevated vₚ in epilepsy aligns with the literature implicating epileptogenic vascular remodeling and angiogenesis, in which angiogenic signaling (notably vascular endothelial growth factor (VEGF)) drives endothelial sprouting and increased microvascular density (Rigau et al., 2007). The increase in metabolic demand during seizures can upregulate VEGF produced by neurons and astrocytes (Van Lanen et al., 2021); VEGF signaling through VEGFR-2 activates pathways (e.g., MEK–MAPK and PI3K) that promote endothelial proliferation, migration, and sprouting of new vessels (Obermeier et al., 2013). In this framework, higher vₚ may reflect microvascular expansion rather than purely transient hemodynamic change, as an indirect imaging marker. Importantly, newly formed vessels can remain immature and more permeable, contributing to BBBD (Van Lanen et al., 2021). The asymmetric overlap between BBBD and vₚ abnormalities is consistent with this framework: only 9.97% of BBBD-abnormal regions also showed elevated vₚ, whereas 37.9% of vₚ-abnormal regions also showed BBBD. Neuronal activity during seizures has also been linked to tight junction protein alterations and extracellular-matrix remodeling through MMP-2/−9 activity (Morin-Brureau et al., 2011; Kim et al., 2015). Human pathology studies further support abnormal microvascular morphology in epileptic tissue (e.g., basement-membrane alterations and string vessels), consistent with dysregulated vessel maturation (Kastanauskaite et al., 2009; Mott et al., 2009). Together with prior evidence of BBBD in DRE (Cafri et al., 2025), the present work adds a complementary intravascular volume readout that may help identify regions where microvascular structural remodeling and functional failure (BBBD) co-occur.

One of our important findings was the combination of increased abnormality burden with limited exact regional overlap across patients. PWE showed more abnormal regions than controls, yet only a small proportion of patients showed abnormal vₚ increases in the same atlas-defined regions. This pattern suggests that vₚ abnormalities were not concentrated in a single recurrent region across the cohort but instead varied across patients while sometimes converging within broader anatomical territories. In line with this heterogeneity, the lack of corrected lobar significance is biologically plausible, as patient-specific abnormalities may cluster within broader territories without recurring in the same lobe consistently enough to produce robust group-level effects. Accordingly, the nominal fronto-temporal and limbic findings from the site-specific lobar analyses should be interpreted as exploratory spatial trends rather than definitive group-level effects. This distinction is important because multiple-comparison correction is required when several tests contribute to a final confirmatory conclusion, whereas exploratory analyses may be useful for generating hypotheses when interpreted descriptively rather than as definitive inference (Bender and Lange, 2001). The less consistent limbic findings may further reflect site-specific cohort composition, as the DAL cohort included only presurgical patients and one generalized epilepsy case, as well as the anatomical heterogeneity of the atlas-defined limbic compartment, which included the amygdala, hippocampus, basal forebrain, cingulate regions, insula, and subcallosal area.

These spatial patterns are consistent with prior experimental evidence. Experimental models of epilepsy have shown increased vessel density and angiogenesis in fronto-temporal and limbic structures, including the amygdala and hippocampus, and have demonstrated that such vascular changes can co-occur with BBB leakage during epileptogenesis (Rigau et al., 2007; Hayward et al., 2010; Marcon et al., 2009). This provides a mechanistic context for the fronto-temporal predominance of vₚ abnormalities observed here.

Importantly, seizure-related vascular remodeling does not necessarily imply physiologically effective perfusion. Abnormal astrocyte-vessel signaling, pericyte dysfunction, and impaired neurovascular coupling may instead produce inefficient vascular responses around epileptogenic tissue (Prager et al., 2019; Tran et al., 2020; Zhao et al., 2011). This is consistent with MRI-based studies showing perfusion abnormalities in and around epileptogenic regions (Farrell et al., 2016; Gupta et al., 2018).

Moreover, our epilepsy type and lesion-related analyses further suggest that vₚ may capture aspects of the spatial organization of abnormality, in addition to overall abnormality burden. Patients with suspected generalized epilepsy showed a higher number of abnormal regions than those with focal epilepsy, but no corresponding increase in voxel-wise abnormality burden. This indicates that, in generalized epilepsy, vₚ abnormalities may be distributed across more anatomical regions without a greater total volume of affected tissue. Because most patients with generalized epilepsy were recruited at SUMC, this cohort composition may also partly explain the stronger region-wise group difference observed at SUMC than at DAL (Fig. S4). This interpretation is consistent with prior literature describing generalized epilepsies as distributed network disorders, with abnormalities involving frontal, broader cortical, and cerebellar networks (Moeller et al., 2011; McGill et al., 2012). Similarly, MRI-positive patients did not show a greater voxel-wise burden of vₚ abnormality than MRI-negative patients, but they did show greater region-wise abnormality burden and more frequent ipsilateral clustering near the lesion. This suggests that lesional epilepsy may be associated with a more spatially organized or focal pattern of vₚ abnormality, rather than a greater overall burden of abnormal vₚ. Pathology studies in focal cortical dysplasia similarly report increased vascular density within and adjacent to the lesion, consistent with the focal cortical dysplasia case illustrated in Fig. 3A (Wintermark et al., 2013). Importantly, this spatial information may have clinical relevance in MRI-negative or diagnostically ambiguous cases, where focal vₚ abnormalities could help refine epilepsy classification or reveal subtle lesions, such as radiologically occult focal cortical dysplasia, that are not apparent on conventional MRI. However, this remains hypothesis-generating and requires validation against presurgical evaluation, histopathology, and surgical outcomes.

In a recent study, we showed that while DCE-MRI reveals BBBD more frequently in PWE compared with healthy controls, BBBD was often diffuse (see Fig. 4A) (Cafri et al., 2025). We suggested that diffuse BBBD may reflect transient microvascular dysfunction due to propagation of epileptic activity or non-related cortical pathology underlying, for example, co-morbidities. In contrast, vₚ abnormalities, likely reflecting more structural changes, were more focal.

The present analysis, however, added the extent of the convergence of vₚ and BBBD abnormalities and asked whether their integration could provide a more spatially specific vascular signature. The directionality analysis showed that joint abnormalities were primarily driven by concurrent elevation of both markers, supporting the interpretation that the vₚ-BBBD overlap identifies regions where structural and functional vascular abnormalities co-occur. Combining structural and functional microvascular pathology revealed a less diffuse and more focal abnormality pattern, closer to the clinically suspected epileptogenic territory in representative patients (Fig. 4A). This increased specificity may provide a hypothesis-generating approach for future studies during pre-surgical evaluation (Anyanwu and Motamedi, 2018; Englot and Chang, 2014; Sheng et al., 2018). The spatial-null sensitivity analysis further supported this pattern by accounting for spatial autocorrelation, where apparent overlap may partly arise because neighboring brain regions are not independent (Alexander-Bloch et al., 2018). Nevertheless, these localization findings remain exploratory because clinically suspected territories were inferred from available presurgical information, qualitative categorization was performed by a single rater, and validation against independent reference standards, including intracranial EEG, surgical outcome, and histopathology, was not available.

Previous machine-learning studies in epilepsy MRI have shown that imaging features derived from structural, diffusion, and radiomic analyses can differentiate people with epilepsy from controls and can also support lesion detection and lateralization of seizure focus (Sone and Beheshti, 2021; Del Gaizo et al., 2017; Park et al., 2020). However, the combination of intravascular plasma volume with BBBD derived from DCE-MRI remains underexplored. Our machine-learning results support the complementary value of integrating structural and functional vascular measures. Performance was reasonably consistent across all 3 classifiers, suggesting that the observed signal was not confined to a single modeling approach. The representation of both z-vₚ and z-BBBD features among the top LR coefficients suggests that these measures may provide complementary information. Notably, SVM yielded the highest ROC-AUC, indicating the strongest overall discrimination between PWE and controls across decision thresholds (Hajian-Tilaki, 2013), whereas LR achieved the highest balanced accuracy, indicating the most effective class-balanced classification on the held-out set (Brodersen et al., 2010). The higher LR held-out BAC relative to training cross-validation should be interpreted in the context of the modest held-out test-set size, possible sampling variability from a single 70/30 split, and bootstrapped confidence intervals, which indicate uncertainty around the exact performance estimates. Therefore, rather than establishing diagnostic utility, these results provide exploratory evidence that combined vascular and barrier features contain valuable discriminative information, warranting further validation in larger, more balanced cohorts alongside conventional MRI-derived features.

A key limitation is that abnormality detection for vₚ relies on the quality and harmonization of the normative control reference. Our control cohort was multi-site with differences in scanner and contrast agent, which may introduce residual variability despite robust MAD-based z-scoring. In addition, the PWE cohort was clinically heterogeneous, with relatively small numbers of patients within each epilepsy subgroup, including generalized, frontal, and temporal epilepsy. Moreover, lesional subgroups were relatively small, and histopathology and postsurgical outcome data were unavailable; therefore, lesion categories were based on clinical impressions rather than confirmed histopathological diagnoses. Medication use represents an additional potential confound. Although no significant differences in vₚ abnormality burden were observed for most commonly used antiseizure medications, lamotrigine use was associated with lower vₚ abnormality burden and should be interpreted cautiously given frequent polytherapy, indication bias, and the exploratory nature of this analysis. Future work should build a larger harmonized normative database for vₚ with site-adjusted modeling and validate the machine-learning findings in larger independent cohorts to further establish model stability, generalizability, and performance across epilepsy subtypes. In addition, conventional MRI-derived features were not incorporated in our machine learning analysis; therefore, future studies should evaluate whether DCE-MRI-derived vascular features provide incremental diagnostic value beyond standard MRI. Extending the approach to white matter, including tract-level mapping and network analyses, may clarify whether DRE involves microvascular remodeling beyond gray matter and further improve patient-specific localization. Finally, the vascular integration localization method can be further validated using more quantitative approaches against surgical outcomes, intracranial EEG, and histopathology.

In summary, our study supports plasma volume fraction (vₚ) as a sensitive DCE-MRI-derived marker of microvascular abnormalities in drug-resistant epilepsy. PWE showed increased vₚ abnormality burden compared with controls, with heterogeneous but regionally organized patterns. Combining vₚ and BBBD reduced the spatial diffuseness of BBBD-only abnormalities and showed preliminary qualitative concordance with clinically suspected epileptogenic territories. These findings support further prospective validation of combined vascular-barrier imaging against intracranial EEG, surgical outcome, and histopathology.

## CRediT authorship contribution statement

**Amirhossein Talebanpour:** Writing – review & editing, Writing – original draft, Visualization, Software, Methodology, Investigation, Formal analysis, Conceptualization. **Sheida Mirloo:** Writing – review & editing, Visualization, Software, Methodology. **Alireza Aleali:** Writing – review & editing, Formal analysis. **Lyna Kamintsky:** Software, Methodology. **Yonatan Serlin:** Writing – review & editing. **Nir Cafri:** Resources. **Ilan Goldberg:** Writing – review & editing, Investigation. **Chris Bowen:** Methodology. **Gal Ben-Arie:** Investigation. **Theodor Rüber:** Methodology. **Felix Benninger:** Writing – review & editing, Resources, Project administration, Investigation, Funding acquisition. **Ben Whatley:** Writing – review & editing, Resources, Project administration, Investigation, Funding acquisition. **Alon Friedman:** Writing – review & editing, Supervision, Project administration, Conceptualization.

## Declaration of competing interest

The authors declare that they have no known competing financial interests or personal relationships that could have appeared to influence the work reported in this paper.

## Data Availability

The data that support the findings of this study are available from the corresponding author upon reasonable request.

## References

[bb0005] Alexander-Bloch A.F., Shou H., Liu S., Satterthwaite T.D., Glahn D.C., Shinohara R.T. (2018). On testing for spatial correspondence between maps of human brain structure and function. Neuroimage.

[bb0010] Anyanwu C., Motamedi G.K. (2018). Diagnosis and surgical treatment of drug-resistant epilepsy. Brain Sci..

[bb0015] Banerjee J., Chandra S.P., Kurwale N., Tripathi M. (2014). Epileptogenic networks and drug-resistant epilepsy: present and future perspectives of epilepsy research-utility for the epileptologist and the epilepsy surgeon. Ann. Indian Acad. Neurol..

[bb0020] Baumgartner C., Koren J.P., Britto-Arias M., Zoche L., Pirker S. (2019). Presurgical epilepsy evaluation and epilepsy surgery. F1000Res.

[bb0025] Bender R., Lange S. (2001). Adjusting for multiple testing—when and how?. J. Clin. Epidemiol..

[bb0030] Berkelmans G.F.N., Read S.H., Gudbjörnsdottir S., Wild S.H., Franzen S., Van Der Graaf Y. (2022). Population median imputation was noninferior to complex approaches for imputing missing values in cardiovascular prediction models in clinical practice. J. Clin. Epidemiol..

[bb0035] Bitzer F., Walger L., Bauer T., Schulte F., Gaertner F.C., Schmitz M. (2024). Higher validity, lower radiation: a new ictal single-photon emission computed tomography framework. Ann. Neurol..

[bb0040] Brodersen K.H., Ong C.S., Stephan K.E., Buhmann J.M. (2010).

[bb0045] Cafri N., Mirloo S., Zarhin D., Kamintsky L., Serlin Y., Alhadeed L. (2025). Imaging blood–brain barrier dysfunction in drug-resistant epilepsy: a multi-center feasibility study. Epilepsia.

[bb0050] Chassidim Y., Veksler R., Lublinsky S., Pell G.S., Friedman A., Shelef I. (2013). Quantitative imaging assessment of blood-brain barrier permeability in humans. Fluids Barriers CNS..

[bb0055] Clement P., Mutsaerts H.J., Václavů L., Ghariq E., Pizzini F.B., Smits M. (2018). Variability of physiological brain perfusion in healthy subjects – a systematic review of modifiers. Considerations for multi-center ASL studies. J. Cereb. Blood Flow Metab..

[bb0060] Del Gaizo J., Mofrad N., Jensen J.H., Clark D., Glenn R., Helpern J. (2017). Using machine learning to classify temporal lobe epilepsy based on diffusion MRI. Brain Behav..

[bb0065] Deoni S.C.L., Peters T.M., Rutt B.K. (2005). High-resolution T1 and T2 mapping of the brain in a clinically acceptable time with DESPOT1 and DESPOT2. Magn. Reson. Med..

[bb0070] Englot D.J., Chang E.F. (2014). Rates and predictors of seizure freedom in resective epilepsy surgery: an update. Neurosurg. Rev..

[bb0075] Fantini S., Sassaroli A., Tgavalekos K.T., Kornbluth J. (2016). Cerebral blood flow and autoregulation: current measurement techniques and prospects for noninvasive optical methods. Neurophotonics.

[bb0080] Farrell J.S., Gaxiola-Valdez I., Wolff M.D., David L.S., Dika H.I., Geeraert B.L. (2016). Postictal behavioural impairments are due to a severe prolonged hypoperfusion/hypoxia event that is COX-2 dependent. Ramirez JM, editor. eLife.

[bb0085] Fisher R.S., Cross J.H., D’Souza C., French J.A., Haut S.R., Higurashi N. (2017). Instruction manual for the ILAE 2017 operational classification of seizure types. Epilepsia.

[bb0090] Gaxiola-Valdez I., Singh S., Perera T., Sandy S., Li E., Federico P. (2017). Seizure onset zone localization using postictal hypoperfusion detected by arterial spin labelling MRI. Brain.

[bb0095] Gorelick P.B., Scuteri A., Black S.E., Decarli C., Greenberg S.M., Iadecola C. (2011). Vascular contributions to cognitive impairment and dementia: a statement for healthcare professionals from the American Heart Association/American Stroke Association. Stroke.

[bb0100] Gupta L, Hofman P a. M, Besseling RMH, Jansen JFA, Backes WH. Abnormal blood oxygen level-dependent fluctuations in focal cortical dysplasia and the perilesional zone: initial findings. AJNR Am. J. Neuroradiol. 2018;39(7):1310–5. doi:10.3174/ajnr.A5684 (PubMed PMID: 29794237; PubMed Central PMCID: PMC7655428). PMC7655428 29794237

[bb0105] Hajian-Tilaki K. (2013). Receiver operating characteristic (ROC) curve analysis for medical diagnostic test evaluation. Caspian J. Intern. Med..

[bb0110] Hayward N.M.E.A., Ndode-Ekane X.E., Kutchiashvili N., Gröhn O., Pitkänen A. (2010). Elevated cerebral blood flow and vascular density in the amygdala after status epilepticus in rats. Neurosci. Lett..

[bb0115] Heinemann U., Kaufer D., Friedman A. (2012). Blood-brain barrier dysfunction, TGFβ signaling, and astrocyte dysfunction in epilepsy. Glia.

[bb0120] Kamintsky L., Beyea S.D., Fisk J.D., Hashmi J.A., Omisade A., Calkin C. (2020). Blood-brain barrier leakage in systemic lupus erythematosus is associated with gray matter loss and cognitive impairment. Ann. Rheum. Dis..

[bb0125] Kastanauskaite A., Alonso-Nanclares L., Blazquez-Llorca L., Pastor J., Sola R.G., DeFelipe J. (2009). Alterations of the microvascular network in sclerotic hippocampi from patients with epilepsy. J. Neuropathol. Exp. Neurol..

[bb0130] Kim J.Y., Ko A.R., Hyun H.W., Kang T.C. (2015). ETB receptor-mediated MMP-9 activation induces vasogenic edema via ZO-1 protein degradation following status epilepticus. Neuroscience.

[bb0135] Kim B.S., Lee S.T., Yun T.J., Lee S.K., Paeng J.C., Jun J. (2016). Capability of arterial spin labeling MR imaging in localizing seizure focus in clinical seizure activity. Eur. J. Radiol..

[bb0140] Kuhn M. (2008). Building predictive models in R using the caret package. J. Stat. Softw..

[bb0145] Leys C., Ley C., Klein O., Bernard P., Licata L. (2013). Detecting outliers: do not use standard deviation around the mean, use absolute deviation around the median. J. Exp. Soc. Psychol..

[bb0150] Marcon J., Gagliardi B., Balosso S., Maroso M., Noé F., Morin M. (2009). Age-dependent vascular changes induced by status epilepticus in rat forebrain: implications for epileptogenesis. Neurobiol. Dis..

[bb0155] McGill M.L., Devinsky O., Kelly C., Milham M., Castellanos F.X., Quinn B.T. (2012). Default mode network abnormalities in idiopathic generalized epilepsy. Epilepsy Behav..

[bb0160] Moeller F., Maneshi M., Pittau F., Gholipour T., Bellec P., Dubeau F. (2011). Functional connectivity in patients with idiopathic generalized epilepsy. Epilepsia.

[bb0165] Morin-Brureau M., Lebrun A., Rousset M.C., Fagni L., Bockaert J., de Bock F. (2011). Epileptiform activity induces vascular remodeling and zonula occludens 1 downregulation in organotypic hippocampal cultures: role of VEGF signaling pathways. J. Neurosci..

[bb0170] Mott R.T., Thore C.R., Moody D.M., Glazier S.S., Ellis T.L., Brown W.R. (2009). Reduced ratio of afferent to total vascular density in mesial temporal sclerosis. J. Neuropathol. Exp. Neurol..

[bb0175] Nagesh C., Kumar S., Menon R., Thomas B., Radhakrishnan A., Kesavadas C. (2018). The imaging of localization related symptomatic epilepsies: the value of arterial spin labelling based magnetic resonance perfusion. Korean J. Radiol..

[bb0180] Neurodebian (2026). GitHub [Internet]. [cited 2026 Feb 1]. spm12/tpm at master · neurodebian/spm12. Available from. https://github.com/neurodebian/spm12/tree/master/tpm.

[bb0185] Obermeier B., Daneman R., Ransohoff R.M. (2013). Development, maintenance and disruption of the blood-brain barrier. Nat. Med..

[bb0190] Park Y.W., Choi Y.S., Kim S.E., Choi D., Han K., Kim H. (2020). Radiomics features of hippocampal regions in magnetic resonance imaging can differentiate medial temporal lobe epilepsy patients from healthy controls. Sci. Rep..

[bb0195] Prager O., Kamintsky L., Hasam-Henderson L.A., Schoknecht K., Wuntke V., Papageorgiou I. (2019). Seizure-induced microvascular injury is associated with impaired neurovascular coupling and blood-brain barrier dysfunction. Epilepsia.

[bb0200] Reiter J.T., Schulte F., Bauer T., David B., Endler C., Isaak A. (2024). Evidence for interictal blood-brain barrier dysfunction in people with epilepsy. Epilepsia.

[bb0205] Rigau V., Morin M., Rousset M.C., De Bock F., Lebrun A., Coubes P. (2007). Angiogenesis is associated with blood-brain barrier permeability in temporal lobe epilepsy. Brain.

[bb0210] Rüber T., David B., Lüchters G., Nass R.D., Friedman A., Surges R. (2018). Evidence for peri-ictal blood–brain barrier dysfunction in patients with epilepsy. Brain.

[bb0215] Schulte F., Reiter J.T., Bauer T., Taube J., Bitzer F., Witt J. (2024). Interictal blood–brain barrier dysfunction in piriform cortex of people with epilepsy. Ann. Clin. Transl. Neurol..

[bb0220] Serlin Y., Ofer J., Ben-Arie G., Veksler R., Ifergane G., Shelef I. (2019). Blood-brain barrier leakage: a new biomarker in transient ischemic attacks. Stroke.

[bb0225] Sheng J., Liu S., Qin H., Li B., Zhang X. (2018). Drug-resistant epilepsy and surgery. Curr. Neuropharmacol..

[bb0230] Sone D., Beheshti I. (2021). Clinical application of machine learning models for brain imaging in epilepsy: a review. Front. Neurosci..

[bb0235] Sourbron S.P., Buckley D.L. (2011). On the scope and interpretation of the Tofts models for DCE-MRI. Magn. Reson. Med..

[bb0240] SPM (2026). Statistical Parametric Mapping [Internet]. https://www.fil.ion.ucl.ac.uk/spm/.

[bb0245] Takahara K., Morioka T., Shimogawa T., Haga S., Kameda K., Arihiro S. (2018). Hemodynamic state of periictal hyperperfusion revealed by arterial spin-labeling perfusion MR images with dual postlabeling delay. eNeurologicalSci.

[bb0250] Tibshirani R. (1996). Regression shrinkage and selection via the Lasso. J. R. Stat. Soc. Series B Stat. Methodology.

[bb0255] Tofts P.S. (1997). Modeling tracer kinetics in dynamic Gd-DTPA MR imaging. Magn. Reson. Imaging.

[bb0260] Tran C.H.T., George A.G., Teskey G.C., Gordon G.R. (2020). Seizures elevate gliovascular unit Ca2+ and cause sustained vasoconstriction. JCI Insight.

[bb0265] van der Kleij L.A., De Vis J.B., de Bresser J., Hendrikse J., Siero J.C.W. (2020). Arterial CO2 pressure changes during hypercapnia are associated with changes in brain parenchymal volume. Eur Radiol Exp..

[bb0270] Van Lanen R.H., Melchers S., Hoogland G., Schijns O.E., Zandvoort M.A.V., Haeren R.H. (2021). Microvascular changes associated with epilepsy: a narrative review. J. Cereb. Blood Flow Metab..

[bb0275] Veksler R., Shelef I., Friedman A. (2014). Blood–brain barrier imaging in human neuropathologies. Arch. Med. Res. Specia Issue: Blood-Brain Barrier in Neurological Diseases.

[bb0280] Veksler R., Vazana U., Serlin Y., Prager O., Ofer J., Shemen N. (2020). Slow blood-to-brain transport underlies enduring barrier dysfunction in American football players. Brain.

[bb0285] Verdi S., Kia S.M., Yong K.X.X., Tosun D., Schott J.M., Marquand A.F. (2023). Revealing individual neuroanatomical heterogeneity in Alzheimer disease using neuroanatomical normative modeling. Neurology.

[bb0290] Vos de Wael R., Benkarim O., Paquola C., Lariviere S., Royer J., Tavakol S. (2020). BrainSpace: a toolbox for the analysis of macroscale gradients in neuroimaging and connectomics datasets. Commun. Biol..

[bb0295] Vu E.L., Brown C.H., Brady K.M., Hogue C.W. (2024). Monitoring of cerebral blood flow autoregulation: physiologic basis, measurement, and clinical implications. Br. J. Anaesth..

[bb0300] Wardlaw J.M., Smith C., Dichgans M. (2019). Small vessel disease: mechanisms and clinical implications. Lancet Neurol..

[bb0305] Wintermark P., Lechpammer M., Warfield S.K., Kosaras B., Takeoka M., Poduri A. (2013). Perfusion imaging of focal cortical dysplasia using arterial spin labeling: correlation with histopathological vascular density. J. Child Neurol..

[bb0310] Zhao M., Nguyen J., Ma H., Nishimura N., Schaffer C.B., Schwartz T.H. (2011). Preictal and ictal neurovascular and metabolic coupling surrounding a seizure focus. J. Neurosci..

