# Supplementary material for: Plasma volume fraction in drug-resistant epilepsy: A DCE-MRI marker of microvascular pathology
Source: Neuroimage Clin. 2026 Jul 27;51:104044. doi: 10.1016/j.nicl.2026.104044 (PMC13452395; doi:10.1016/j.nicl.2026.104044)
Supplement: Supplementary file 1 [file mmc1.docx]

**SUPPLEMENTARY MATERIALS**

FIGURE S1.

**FIGURE S1. Average T1 relaxation values across sites.** Average T1 relaxation values are shown to illustrate site-related differences in T1, which can influence DCE-MRI signal conversion and raw vₚ estimation. These differences supported the use of site-specific control-referenced modified z-scores for inter-site abnormality analyses. T1, longitudinal relaxation time; DCE-MRI, dynamic contrast-enhanced magnetic resonance imaging; vₚ, plasma volume fraction.

FIGURE S2.

**FIGURE S2. Regional distribution of elevated vₚ abnormalities in PWE.** Frequency map showing the percentage of PWE with suprathreshold vₚ abnormality (z-vₚ > 2) across the 120 atlas-defined regions. Warmer colors indicate regions more frequently abnormal across patients. PWE, people with epilepsy; vₚ, plasma volume fraction.

FIGURE S3.

B.

A.

**FIGURE S3. vₚ abnormality burden by epilepsy phenotype.** A. Region-wise abnormality burden, quantified as the number of atlas regions with z-vₚ > 2, was higher in generalized epilepsy than focal epilepsy (median ± MAD: 18 ± 9.5 vs 7 ± 7.5; n = 10 vs 39; *p* = 0.0095). B. Voxel-wise abnormality burden, quantified as the percentage of brain voxels with z-vₚ > 2, did not differ between generalized and focal epilepsy (median ± MAD: 14.19% ± 12.5 vs 10.04% ± 5.14; *p* = 0.5581). Voxel-wise burden was quantified as the percentage of brain voxels with z-vₚ > 2, and region-wise burden as the number of atlas regions with z-vₚ > 2. Points represent individual subjects. Dashed horizontal lines indicate the median and interquartile range. Two-sided Mann–Whitney U tests were used. vₚ, plasma volume fraction; MAD, median absolute deviation.

FIGURE S4.

A.

B.

D.

C.

**FIGURE S4. Site-matched vₚ abnormality burden comparisons.** PWE were compared with their corresponding site-specific control cohorts separately for SUMC and DAL. A. In SUMC, voxel-wise abnormality burden did not differ significantly between PWE (n=28) and controls (n=43) (median [IQR]: 13.44 [5.579–23.54] vs 8.399 [3.952–12.18]; *p* = 0.0915). B. In SUMC, region-wise abnormality burden was higher in PWE (n=28) than controls (n=43) (median [IQR]: 19.00 [12.00–26.50] vs 7.00 [4.00–10.00]; *p*<0.0001 ). C. In DAL, voxel-wise abnormality burden did not differ significantly between PWE and controls (median [IQR]: 8.867 [5.634–15.57] vs 6.810 [3.417–11.63]; *p* = 0.0930). D. In DAL, region-wise abnormality burden did not differ significantly between PWE and controls (median [IQR]: 4.00 [1.50–6.00] vs 2.00 [1.00–7.00]; *p* = 0.4546). Voxel-wise burden was quantified as the percentage of brain voxels with z-vₚ > 2, and region-wise burden as the number of atlas regions with z-vₚ > 2. Points represent individual subjects. Dashed horizontal lines indicate the median and interquartile range. Two-sided Mann–Whitney U tests were used. PWE, people with epilepsy; vₚ, plasma volume fraction; IQR, interquartile range; SUMC, Soroka Medical Center; DAL, Dalhousie.

FIGURE S5.

**FIGURE S5. Feature importance from the logistic regression classifier.** Features retained after L1-based selection are shown according to their relative contribution to the final logistic regression model distinguishing PWE from controls. Both z-vₚ and z-BBBD features contributed to model classification. PWE, people with epilepsy; vₚ, plasma volume fraction; BBBD, blood-brain barrier dysfunction.

FIGURE S6.

**FIGURE S6. Confusion matrices for the logistic regression classifier.** Training and held-out test set confusion matrices are shown for the LR model used to distinguish PWE from controls based on regional z-vₚ and z-BBBD features. PWE, people with epilepsy; LR, logistic regression; vₚ, plasma volume fraction; BBBD, blood-brain barrier dysfunction.

Figure S7.

**FIGURE S7. Spatial-null sensitivity analysis.** Representative examples illustrate the clinically suspected epileptogenic territory, the regional joint vascular abnormality map, and their spatial overlap for three focal epilepsy patients. Patient 1: suspected parietal epilepsy, female, 32 years; Patient 2: frontal/midline epilepsy, male, 29 years; Patient 3: temporal lobe epilepsy, male, 23 years. The observed maps are shown in the first row, followed by three representative Moran spectral randomization null maps. Blue indicates the clinically suspected epilepsy region, orange indicates regions with co-occurring suprathreshold vₚ and BBBD abnormalities (z-vₚ > 2 ∩ z-BBBD > 2), and purple indicates spatial overlap. The histogram shows the distribution of localizing rates obtained across 1,000 spatially constrained null permutations, with the blue dashed line indicating the mean null localizing rate, gray dashed lines indicating the 95% null interval, and the purple dashed line indicating the observed localizing rate from the qualitative analysis. The observed localizing rate was 71.8% (28/39 focal epilepsy cases), compared with a mean null localizing rate of 51.0% and a 95% null interval of 41.0–61.5% (empirical *p* = 0.001). BBBD, blood-brain barrier dysfunction; vₚ, plasma volume fraction.

TABLE S1.

| Patient No. | Focal/ Generalized | Suspected Epilepsy Regions | Clinical Diagnosis | MRI Findings |
| --- | --- | --- | --- | --- |
| 1 | Focal | Rt. Temporal lobe | TLE | No visible lesion |
| 2 | Focal | Frontal lobe | Susp. FLE | No visible lesion |
| 3 | Focal | Rt. Frontal lobe | FLE | No visible lesion |
| 4 | Focal | Parietal | Focal epilepsy,  Suspected limbic onset | No visible lesion |
| 5 | Focal | Frontal lobe | Susp. FLE | No visible lesion |
| 6 | Focal | Bifrontal, Midline | FLE | No visible lesion |
| 7 | Focal | Lt. Temporal lobe | TLE | No visible lesion |
| 8 | Focal | Limbic | Focal epilepsy, suspected limbic onset | No visible lesion |
| 9 | Focal | Frontal lobe | FLE | No visible lesion |
| 10 | Focal | Frontal lobe | FLE | Heterotropia & Schizencephaly Rt. Parietal |
| 11 | Focal | Lt. Temporal lobe | TLE | No visible lesion |
| 12 | Focal | Lt. Temporal lobe | TLE | No visible lesion |
| 13 | Focal | Rt. Temporal lobe | TLE | No visible lesion |
| 14 | Focal | Temporal lobe | Susp. TLE | Mesial temporal sclerosis, bilateral |
| 15 | Focal | Lt. Temporal lobe | TLE | No visible lesion |
| 16 | Focal | Parietal | TLE | No visible lesion |
| 17 | Focal | Lt. Temporal lobe | TLE | No visible lesion |
| 18 | Generalized | Generalized/ non-focal | Generalized epilepsy | White matter lesion (Rt. Superior frontal) |
| 19 | Focal | Rt. Temporal lobe | TLE | No visible lesion |
| 20 | Focal | Lt. Temporal lobe | TLE | No visible lesion |
| 21 | Focal | Frontal lobe | Susp. FLE | Tiny hemosiderin focus in the left occipital region; significance is questionable. |
| 22 | Focal | Rt. Temporal lobe | TLE | Rt. mesial temporal sclerosis |
| 23 | Focal | Frontal lobe | Susp. FLE | No visible lesion |
| 24 | Focal | Lt. Temporal lobe | TLE | No visible lesion |
| 25 | Focal | Frontal lobe | FLE | Gliotic changes, post removal of AVM, Rt. Parietal |
| 26 | Generalized | Generalized/ non-focal | Generalized epilepsy | No visible lesion |
| 27 | Generalized | Generalized/ non-focal | Generalized epilepsy | No visible lesion |
| 28 | Focal | Temporal lobe | TLE | Old cortical gliotic focus in the right occipital region. Rt. MTS. |
| 29 | Focal | Rt. temporal & Lt. Occipital | Focal epilepsy | No visible lesion |
| 30 | Focal | Temporal lobe | TLE | Old Infarct (Lt. basal nuclei) with white matter changes |
| 31 | Generalized | Generalized/ non-focal | Generalized epilepsy | No visible lesion |
| 32 | Focal | Frontal lobe | Nocturnal frontal lobe epilepsy (NFLE) | Frontal cortical dysplasia |
| 33 | Generalized | Generalized/ non-focal | Generalized epilepsy | No visible lesion |
| 34 | Generalized | Generalized/ non-focal | JME | No visible lesion |
| 35 | Generalized | Generalized/ non-focal | Generalized epilepsy | Temporal lobe cavernoma, Lt. |
| 36 | Focal | Limbic | Focal epilepsy, suspected limbic onset | No visible lesion |
| 37 | Focal | Lt. Frontal lobe | Multi focal epilepsy Susp. FLE | Cortical malformation, Lt. Frontal |
| 38 | Focal | Left hemisphere | Focal epilepsy | No visible lesion |
| 39 | Focal | Rt. Frontal, Midline | FLE | No visible lesion |
| 40 | Focal | Bitemporal lobe | TLE | No visible lesion |
| 41 | Focal | Bitemporal lobe | TLE | Bi-mesial temporal sclerosis |
| 42 | Focal | Lt. Temporal lobe | TLE | No visible lesion |
| 43 | Generalized | Generalized/ non-focal | Generalized epilepsy | No visible lesion |
| 44 | Focal | Temporal lobe | TLE | Post-surgical removal of cavernoma, Lt. Parietal |
| 45 | Focal | Lt. Frontal lobe | Focal epilepsy | Small vessel disease, bilateral |
| 46 | Generalized | Generalized/ non-focal | Generalized epilepsy | Focal hemosiderin, Lt. occipital & parietal |
| 47 | Focal | Lt. Fronto-Temporal | Lt. Frontal/Temporal Epilepsy | No visible lesion |
| 48 | Generalized | Generalized/ non-focal | JME | No visible lesion |
| 49 | Focal | Lt. Temporal lobe | TLE | Lt. mesial temporal sclerosis |

TABLE S2.

| Region | Pct |
| --- | --- |
| Right OrIFG orbital part of the inferior frontal gyrus | 36.735 |
| Left MTG middle temporal gyrus | 34.694 |
| Left CO central operculum | 32.653 |
| Right OpIFG opercular part of the inferior frontal gyrus | 32.653 |
| Right PoG postcentral gyrus | 32.653 |
| Right PT planum temporale | 32.653 |
| Right IOG inferior occipital gyrus | 28.571 |
| Right MFG middle frontal gyrus | 28.571 |
| Left OFuG occipital fusiform gyrus | 28.571 |
| Left Ent entorhinal area | 26.531 |
| Right ITG inferior temporal gyrus | 26.531 |
| Right LiG lingual gyrus | 26.531 |
| Left POrG posterior orbital gyrus | 26.531 |
| Right PrG precentral gyrus | 26.531 |
| Right CO central operculum | 24.49 |
| Left IOG inferior occipital gyrus | 24.49 |
| Left OrIFG orbital part of the inferior frontal gyrus | 24.49 |
| Left PCgG posterior cingulate gyrus | 24.49 |
| Right PIns posterior insula | 24.49 |
| Right PO parietal operculum | 24.49 |
| Right PP planum polare | 24.49 |
| Left SPL superior parietal lobule | 24.49 |
| Left STG superior temporal gyrus | 24.49 |
| Right Caudate | 22.449 |
| Left Cerebellum Exterior | 22.449 |
| Right Cun cuneus | 22.449 |
| Right FuG fusiform gyrus | 22.449 |
| Left ITG inferior temporal gyrus | 22.449 |
| Left LOrG lateral orbital gyrus | 22.449 |
| Left MOrG medial orbital gyrus | 22.449 |
| Right MSFG superior frontal gyrus medial segment | 22.449 |
| Left PCu precuneus | 22.449 |
| Left PoG postcentral gyrus | 22.449 |
| Right SMC supplementary motor cortex | 22.449 |
| Right Cerebellum White Matter | 20.408 |
| Left Basal Forebrain | 20.408 |
| Left Cun cuneus | 20.408 |
| Right OFuG occipital fusiform gyrus | 20.408 |
| Left PP planum polare | 20.408 |
| Left SMG supramarginal gyrus | 20.408 |
| Right TrIFG triangular part of the inferior frontal gyrus | 20.408 |
| Right Amygdala | 18.367 |
| Left Caudate | 18.367 |
| Right Thalamus Proper | 18.367 |
| Right AnG angular gyrus | 18.367 |
| Left LiG lingual gyrus | 18.367 |
| Right MCgG middle cingulate gyrus | 18.367 |
| Left MFG middle frontal gyrus | 18.367 |
| Right MOrG medial orbital gyrus | 18.367 |
| Left AOrG anterior orbital gyrus | 16.327 |
| Left AnG angular gyrus | 16.327 |
| Right PCu precuneus | 16.327 |
| Right PHG parahippocampal gyrus | 16.327 |
| Left PO parietal operculum | 16.327 |
| Left PrG precentral gyrus | 16.327 |
| Left PT planum temporale | 16.327 |
| Right SFG superior frontal gyrus | 16.327 |
| Right Putamen | 14.286 |
| Cerebellar Vermal Lobules VIII-X | 14.286 |
| Right Basal Forebrain | 14.286 |
| Right AIns anterior insula | 14.286 |
| Left AIns anterior insula | 14.286 |
| Right Calc calcarine cortex | 14.286 |
| Right MOG middle occipital gyrus | 14.286 |
| Left MPrG precentral gyrus medial segment | 14.286 |
| Left PIns posterior insula | 14.286 |
| Left SFG superior frontal gyrus | 14.286 |
| Left SMC supplementary motor cortex | 14.286 |
| Left Accumbens Area | 12.245 |
| Left Cerebellum White Matter | 12.245 |
| Cerebellar Vermal Lobules VI-VII | 12.245 |
| Left ACgG anterior cingulate gyrus | 12.245 |
| Left Calc calcarine cortex | 12.245 |
| Right FRP frontal pole | 12.245 |
| Left MCgG middle cingulate gyrus | 12.245 |
| Left MFC medial frontal cortex | 12.245 |
| Left OpIFG opercular part of the inferior frontal gyrus | 12.245 |
| Left TMP temporal pole | 12.245 |
| Right TTG transverse temporal gyrus | 12.245 |
| Left TTG transverse temporal gyrus | 12.245 |
| Brain Stem | 10.204 |
| Right Hippocampus | 10.204 |
| Left Putamen | 10.204 |
| Cerebellar Vermal Lobules I-V | 10.204 |
| Right FO frontal operculum | 10.204 |
| Left FuG fusiform gyrus | 10.204 |
| Right MFC medial frontal cortex | 10.204 |
| Left MOG middle occipital gyrus | 10.204 |
| Left Amygdala | 8.1633 |
| Left Ventral DC | 8.1633 |
| Right Ent entorhinal area | 8.1633 |
| Right MPoG postcentral gyrus medial segment | 8.1633 |
| Left MPoG postcentral gyrus medial segment | 8.1633 |
| Right MPrG precentral gyrus medial segment | 8.1633 |
| Left FRP frontal pole | 6.1224 |
| Right SCA subcallosal area | 6.1224 |
| Left SCA subcallosal area | 6.1224 |
| Right SOG superior occipital gyrus | 6.1224 |
| Right Accumbens Area | 4.0816 |
